# Bis(methyl­sulfon­yl)methane

**DOI:** 10.1107/S1600536814016201

**Published:** 2014-07-23

**Authors:** Riad Awad, Eyad Mallah, Wael Abu Dayyih, Kamal Sweidan, Manfred Steimann

**Affiliations:** a Faculty of Pharmacy and Medical Science, University of Petra, Amman, Jordan; b Department of Chemistry, Faculty of Science, The University of Jordan, Amman, Jordan; c University of Tübingen, Inorganic Chemistry, Auf der Morgenstelle 18, 72076 Tübingen, Germany

**Keywords:** crystal structure

## Abstract

In the title compound, C_3_H_8_O_4_S_2_, the two central S—C(H_2_) bond lengths are almost identical [1.781 (2) and 1.789 (2) Å]. In the crystal, each mol­ecule utilizes CH_2_ and CH_3_ bonds to form weak C—H⋯O hydrogen bonds to six other mol­ecules, thus linking mol­ecules into a three-dimensional network.

## Related literature   

For the structures of similar compounds, see: Berthou *et al.* (1972 ▶); Glidewell *et al.* (1995 ▶, 1996 ▶); Meehan *et al.* (1997 ▶); Zhang *et al.* (2009 ▶). For information of the use of the title compound in the food industry, see: Awaleh *et al.* (2007 ▶); Gereben & Pusztai (2012 ▶). 
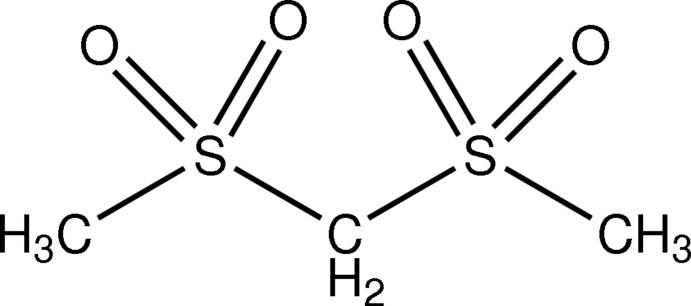


## Experimental   

### 

#### Crystal data  

C_3_H_8_O_4_S_2_
*M* *_r_* = 172.21
Monoclinic, 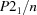

*a* = 11.0496 (18) Å
*b* = 5.793 (3) Å
*c* = 11.0496 (6) Å
β = 96.77 (3)°
*V* = 702.3 (3) Å^3^
*Z* = 4
Mo *K*α radiation
μ = 0.70 mm^−1^
*T* = 173 K
0.25 × 0.05 × 0.05 mm

#### Data collection  

Stoe IPDS diffractometer
9692 measured reflections
1441 independent reflections
1274 reflections with *I* > 2σ(*I*)
*R*_int_ = 0.074

#### Refinement  

*R*[*F* ^2^ > 2σ(*F* ^2^)] = 0.031
*wR*(*F* ^2^) = 0.073
*S* = 1.10
1441 reflections
115 parameters
All H-atom parameters refined
Δρ_max_ = 0.42 e Å^−3^
Δρ_min_ = −0.28 e Å^−3^

### 

Data collection: *IPDS* (Stoe & Cie, 2008 ▶); cell refinement: *X-AREA* (Stoe & Cie, 2008 ▶); data reduction: *IPDS*; program(s) used to solve structure: *SHELXTL* (Sheldrick, 2008 ▶); program(s) used to refine structure: *SHELXTL*; molecular graphics: *SHELXTL*; software used to prepare material for publication: *SHELXTL*.

## Supplementary Material

Crystal structure: contains datablock(s) I, New_Global_Publ_Block. DOI: 10.1107/S1600536814016201/cv5464sup1.cif

Structure factors: contains datablock(s) I. DOI: 10.1107/S1600536814016201/cv5464Isup2.hkl

Supporting information file. DOI: 10.1107/S1600536814016201/cv5464Isup3.cml

CCDC reference: 1013637

Additional supporting information:  crystallographic information; 3D view; checkCIF report

## Figures and Tables

**Table 1:** Hydrogen-bond geometry (Å, °)

*D*—H⋯*A*	*D*—H	H⋯*A*	*D*⋯*A*	*D*—H⋯*A*
C1—H1*A*⋯O4^i^	0.94 (2)	2.55 (2)	3.342 (3)	142.3 (18)
C1—H1*B*⋯O4^ii^	0.92 (2)	2.43 (2)	3.254 (3)	149.1 (19)
C2—H2*C*⋯O5^iii^	0.89 (3)	2.51 (3)	3.365 (3)	160.3 (19)
C3—H3*A*⋯O6^iv^	0.96 (2)	2.56 (2)	3.339 (3)	138.1 (18)
C3—H3*A*⋯O7^v^	0.96 (2)	2.45 (2)	3.206 (3)	135.5 (18)
C3—H3*B*⋯O6^vi^	0.96 (2)	2.27 (2)	3.184 (3)	159.2 (19)

Symmetry codes: (i) 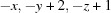; (ii) 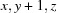; (iii) 
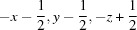
; (iv) 
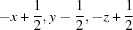
; (v) 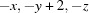; (vi) 
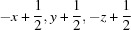
.

**Fig. 1.**
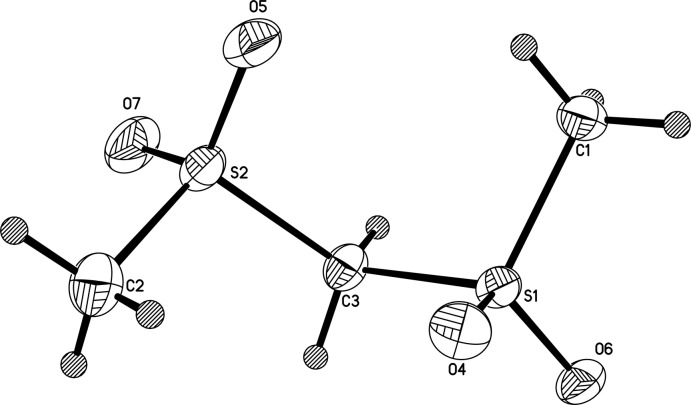
The molecular structure of (I) showing the atomic numbering and 50% probability displacement ellipsoids.

## supplementary crystallographic information

### S1. Comment

The title compound, bis (methylthio) methane (I), is an odorous constituent of truffle which considered as an essential food and industrial flavor used as a primary aromatic ingredient in the truffle oil when combined in an olive oil base (Gereben & Pusztai, 2012; Awaleh *et al.*, 2007).

In (I) (Fig. 1), the two central C—S bond distances [1.781 (2) Å and 1.789 (2) Å] are very close to those reported by Glidewell *et al.* (1995) for (PhSO_2_)_2_CH_2_ [1.786 Å], but smaller than the corresponding distances in the (PhSO_2_)_2_CBr_2_ [1.863 Å] and (PhSO_2_)_2_CI)_2_ [1.854 Å], respectively. This fact could be attributed due to the large size of halogen atoms Br and I relative to the hydrogen atom in the prepared molecule. The S2—C3—S1 angle of 117.20 (10)° and the two O—S—O angles of 118.02 (8)° and 108.72 (9)° entirely consistent with those reported previously by Lucchi *et al.* (1985). The overall conformation is close to the corresponding conformations reported for similar compounds (Berthou *et al.*, 1972; Glidewell *et al.*, 1995).

### S2. Experimental

The title compound was prepared by addition of Bis (methylthio) methane (2.00 ml, 19.58 mmol) to a solution containing acetic acid (16.00 ml, 279.76 mmol) with stirring at 0°C for 15 min. After that (17.00 ml, 720 mmol) of hydrogen peroxide was added drop wise at room temperature, and then the mixture was heated for 3 h at 55°C, the whit precipitate was formed, washed with methanol and dried *in vacuo*. Yield after recystallization from dichloromethane / diethyl ether 2.72 g (82%), as colorless plate crystals.

### S3. Refinement

H atoms were found on electron density map and isotropically refined.

### Crystal data

**Table:**

C_3_H_8_O_4_S_2_	*F*(000) = 360
*M**_r_* = 172.21	*D*_x_ = 1.629 Mg m^−^^3^
Monoclinic, *P*2_1_/*n*	Mo *K*α radiation, λ = 0.71073 Å
Hall symbol: -P 2yn	Cell parameters from 25 reflections
*a* = 11.0496 (18) Å	θ = 5.7–16.2°
*b* = 5.793 (3) Å	µ = 0.70 mm^−^^1^
*c* = 11.0496 (6) Å	*T* = 173 K
β = 96.77 (3)°	Needle, colourless
*V* = 702.3 (3) Å^3^	0.25 × 0.05 × 0.05 mm
*Z* = 4	

### Data collection

**Table:**

Stoe IPDS diffractometer	1274 reflections with *I* > 2σ(*I*)
Radiation source: fine-focus sealed tube	*R*_int_ = 0.074
Graphite monochromator	θ_max_ = 26.3°, θ_min_ = 3.7°
πhi scans	*h* = −13→13
9692 measured reflections	*k* = −6→7
1441 independent reflections	*l* = −13→13

### Refinement

**Table:**

Refinement on *F*^2^	Secondary atom site location: difference Fourier map
Least-squares matrix: full	Hydrogen site location: inferred from neighbouring sites
*R*[*F*^2^ > 2σ(*F*^2^)] = 0.031	All H-atom parameters refined
*wR*(*F*^2^) = 0.073	*w* = 1/[σ^2^(*F*_o_^2^) + (0.0285*P*)^2^ + 0.429*P*] where *P* = (*F*_o_^2^ + 2*F*_c_^2^)/3
*S* = 1.10	(Δ/σ)_max_ = 0.001
1441 reflections	Δρ_max_ = 0.42 e Å^−^^3^
115 parameters	Δρ_min_ = −0.28 e Å^−^^3^
0 restraints	Extinction correction: *SHELXTL* (Sheldrick, 2008), Fc^*^=kFc[1+0.001xFc^2^λ^3^/sin(2θ)]^-1/4^
Primary atom site location: structure-invariant direct methods	Extinction coefficient: 0.0073 (18)

### Special details

**Table:**

Geometry. All e.s.d.'s (except the e.s.d. in the dihedral angle between two l.s. planes) are estimated using the full covariance matrix. The cell e.s.d.'s are taken into account individually in the estimation of e.s.d.'s in distances, angles and torsion angles; correlations between e.s.d.'s in cell parameters are only used when they are defined by crystal symmetry. An approximate (isotropic) treatment of cell e.s.d.'s is used for estimating e.s.d.'s involving l.s. planes.
Refinement. Refinement of *F*^2^ against ALL reflections. The weighted *R*-factor *wR* and goodness of fit *S* are based on *F*^2^, conventional *R*-factors *R* are based on *F*, with *F* set to zero for negative *F*^2^. The threshold expression of *F*^2^ > σ(*F*^2^) is used only for calculating *R*-factors(gt) *etc*. and is not relevant to the choice of reflections for refinement. *R*-factors based on *F*^2^ are statistically about twice as large as those based on *F*, and *R*- factors based on ALL data will be even larger.

### Fractional atomic coordinates and isotropic or equivalent isotropic displacement parameters (Å^2^)

**Table:**

	*x*	*y*	*z*	*U*_iso_*/*U*_eq_	
S1	0.16353 (4)	1.01190 (7)	0.35533 (4)	0.01773 (15)	
S2	−0.07227 (4)	1.00358 (8)	0.18190 (4)	0.02323 (16)	
C1	0.11691 (19)	1.2396 (3)	0.44401 (18)	0.0233 (4)	
H1A	0.033 (2)	1.226 (4)	0.448 (2)	0.034 (6)*	
H1B	0.137 (2)	1.376 (4)	0.409 (2)	0.030 (6)*	
H1C	0.160 (2)	1.220 (4)	0.524 (2)	0.037 (6)*	
C2	−0.0862 (2)	0.7028 (4)	0.1665 (2)	0.0349 (5)	
H2A	−0.052 (2)	0.634 (5)	0.239 (2)	0.041 (7)*	
H2B	−0.046 (3)	0.657 (5)	0.099 (3)	0.048 (7)*	
H2C	−0.166 (3)	0.673 (5)	0.158 (2)	0.049 (8)*	
C3	0.08763 (16)	1.0559 (3)	0.20496 (16)	0.0210 (4)	
H3A	0.127 (2)	0.955 (4)	0.153 (2)	0.028 (6)*	
H3B	0.103 (2)	1.214 (4)	0.1858 (19)	0.027 (6)*	
O4	0.12487 (12)	0.7950 (2)	0.40149 (12)	0.0254 (3)	
O5	−0.12516 (12)	1.0780 (3)	0.28823 (13)	0.0325 (4)	
O6	0.29086 (11)	1.0408 (2)	0.34274 (12)	0.0248 (3)	
O7	−0.11405 (14)	1.1120 (3)	0.06711 (13)	0.0360 (4)	

### Atomic displacement parameters (Å^2^)

**Table:**

	*U*^11^	*U*^22^	*U*^33^	*U*^12^	*U*^13^	*U*^23^
S1	0.0156 (2)	0.0170 (2)	0.0206 (2)	0.00100 (16)	0.00237 (15)	0.00051 (16)
S2	0.0151 (2)	0.0293 (3)	0.0248 (3)	0.00225 (17)	0.00043 (17)	−0.00117 (18)
C1	0.0216 (9)	0.0220 (9)	0.0267 (9)	0.0008 (7)	0.0044 (7)	−0.0055 (8)
C2	0.0240 (11)	0.0325 (12)	0.0479 (14)	−0.0070 (9)	0.0031 (10)	−0.0062 (10)
C3	0.0170 (8)	0.0243 (9)	0.0218 (9)	−0.0013 (7)	0.0024 (7)	0.0023 (7)
O4	0.0295 (7)	0.0196 (6)	0.0273 (7)	−0.0003 (5)	0.0047 (5)	0.0038 (5)
O5	0.0189 (7)	0.0465 (9)	0.0327 (8)	0.0046 (6)	0.0053 (5)	−0.0066 (7)
O6	0.0145 (6)	0.0274 (7)	0.0325 (7)	0.0017 (5)	0.0019 (5)	−0.0021 (6)
O7	0.0273 (7)	0.0492 (10)	0.0292 (7)	0.0091 (7)	−0.0061 (6)	0.0039 (7)

### Geometric parameters (Å, º)

**Table:**

S1—O6	1.4398 (13)	S2—O5	1.4386 (14)
S1—O4	1.4397 (14)	S2—O7	1.4416 (15)
S1—C1	1.7563 (19)	S2—C2	1.756 (2)
S1—C3	1.7889 (18)	S2—C3	1.7811 (19)
			
O6—S1—O4	118.02 (8)	O5—S2—C2	109.70 (11)
O6—S1—C1	108.72 (9)	O7—S2—C2	109.39 (11)
O4—S1—C1	109.82 (10)	O5—S2—C3	108.90 (9)
O6—S1—C3	104.48 (8)	O7—S2—C3	105.13 (9)
O4—S1—C3	109.10 (8)	C2—S2—C3	104.87 (10)
C1—S1—C3	105.96 (10)	S2—C3—S1	117.20 (10)
O5—S2—O7	117.99 (9)		

### Hydrogen-bond geometry (Å, º)

**Table:**

*D*—H···*A*	*D*—H	H···*A*	*D*···*A*	*D*—H···*A*
C1—H1*A*···O4^i^	0.94 (2)	2.55 (2)	3.342 (3)	142.3 (18)
C1—H1*B*···O4^ii^	0.92 (2)	2.43 (2)	3.254 (3)	149.1 (19)
C2—H2*C*···O5^iii^	0.89 (3)	2.51 (3)	3.365 (3)	160.3 (19)
C3—H3*A*···O6^iv^	0.96 (2)	2.56 (2)	3.339 (3)	138.1 (18)
C3—H3*A*···O7^v^	0.96 (2)	2.45 (2)	3.206 (3)	135.5 (18)
C3—H3*B*···O6^vi^	0.96 (2)	2.27 (2)	3.184 (3)	159.2 (19)

Symmetry codes: (i) −*x*, −*y*+2, −*z*+1; (ii) *x*, *y*+1, *z*; (iii) −*x*−1/2, *y*−1/2, −*z*+1/2; (iv) −*x*+1/2, *y*−1/2, −*z*+1/2; (v) −*x*, −*y*+2, −*z*; (vi) −*x*+1/2, *y*+1/2, −*z*+1/2.
